# Potentially toxic elements (PTEs) and ecological risk at waste disposal sites: An analysis of sanitary landfills

**DOI:** 10.1371/journal.pone.0303272

**Published:** 2024-05-17

**Authors:** Anna Podlasek, Magdalena Daria Vaverková, Aleksandra Jakimiuk, Eugeniusz Koda

**Affiliations:** 1 Department of Revitalization and Architecture, Institute of Civil Engineering, Warsaw University of Life Sciences–SGGW, Warsaw, Poland; 2 Department of Applied and Landscape Ecology, Faculty of AgriSciences, Mendel University in Brno, Brno, Czech Republic; Balochistan University of Information Technology Engineering and Management Sciences, PAKISTAN

## Abstract

This study presents an analysis of soil contamination caused by Ni, Zn, Cd, Cu, and Pb at municipal solid waste (MSW) landfills, with a focus on ecological risk assessment. The approach aims to assess how different landfill practices and environmental conditions affect soil contamination with potentially toxic elements (PTEs) and associated environmental risks. Soil samples were collected from MSW landfills in Poland and the Czech Republic. The research included a comprehensive assessment of PTEs in soils in the context of global environmental regulations. The degree of soil contamination by PTEs was assessed using indices: Geoaccumulation Index (I_geo_), Single Pollution Index (Pi), Nemerow Pollution Index (PN), and Load Capacity of a Pollutant (PLI). The ecological risk was determined using the Risk of PTEs (ERi) and Sum of Individual Potential Risk Factors (ERI). The maximum values of the indicators observed for the Radiowo landfill were as follows: I_geo_ = 4.04 for Cd, P_i_ = 24.80 for Cd, PN = 18.22 for Cd, PLI = 2.66, ER_i_ = 744 for Cd, ERI = 771.80. The maximum values of the indicators observed for the Zdounky landfill were as follows: I_geo_ = 1.04 for Cu, P_i_ = 3.10 for Cu, PN = 2.52 for Cu, PLI = 0.27, ER_i_ = 25 for Cd, ERI = 41.86. The soils of the tested landfills were considered to be non-saline, with electrical conductivity (EC) values less than 2,000 μS/cm. Varying levels of PTEs were observed, and geostatistical analysis highlighted hotspots indicating pollution sources. Elevated concentrations of Cd in the soil indicated potential ecological risks. Concentrations of Cu and lead Pb were well below the thresholds set by the environmental legislation in several countries. In addition, Ni concentrations in the soils of both landfills indicated that the average levels were within acceptable limits. Principal Component Analysis (PCA) revealed common sources of PTEs. The identification of specific risk points at the Radiowo and Zdounky sites contributes to a better understanding of potential hazards in landfill environments. By establishing buffer zones and implementing regular maintenance programs, emerging environmental problems can be addressed in a timely manner.

## Introduction

Municipal solid waste (MSW) landfills are recognized as important sources of potentially toxic elements (PTEs) in soils due to the storage of various waste materials, including electronic equipment, batteries, paints, lamps, leather, rubber, and other discarded products containing PTEs [1, 2]. The distribution of PTEs in soils surrounding MSW landfills is influenced by several factors, such as the type and amount of waste disposed [3], the age of the landfill [4], waste management (WM) practices [5], local conditions [6], and physicochemical factors [7]. In addition, the degradation of MSW in landfills and the management of leachate are critical issues that can lead to the release of PTEs into the environment, posing risks to nearby soils and water bodies [8–12]. Contamination of underlying soils and groundwater with PTEs can occur due to the lack of containment measures, particularly in older landfills that lack adequate liners and leachate collection systems [13, 14]. Biogas emissions from landfills also contribute to air pollution with potential adverse health and environmental effects [15].

In addition, precipitation and climatic conditions play a crucial role in the generation of leachate and the transport of PTEs within and around the landfill [16–18]. Increased precipitation can enhance the leaching and runoff of PTEs, thereby increasing the potential for environmental contamination [19]. Soil contamination by PTEs is a challenging problem with significant implications for ecosystems, agriculture, and human activities [20–23]. PTEs adversely affect soil quality by harming microorganisms [24], disrupting nutrient cycling [25], reducing biodiversity [26, 27], and inhibiting plant growth, thereby affecting agricultural productivity [28]. Soil contamination by PTEs also affects drinking water resources and aquatic ecosystems and poses health risks through the bioaccumulation of PTEs in the food chain [29–31]. Soil contamination by PTEs can lead to severe land use restrictions [32], as the affected areas may have to be excluded from residential, agricultural, or recreational use [33]. Therefore, it is essential to adapt competent WM, pollution prevention measures, and appropriate remediation strategies to counteract the adverse effects of PTEs on the environment [34–36]. It is also crucial to recognize that the reduction of PTEs in soils is essential for the achievement of several Sustainable Development Goals, especially those related to health, environmental protection, and responsible resource management [37, 38].

The occurrence of PTEs in soil is often closely linked to various sources of pollution resulting from human activities [39]. Not only waste management facilities but also road traffic can significantly influence the accumulation of PTEs in soils and plants. Metallic particles released during fuel combustion and other chemical compounds present in exhaust gases can be deposited on the soil along roads, leading to the gradual accumulation of PTEs in these areas [40, 41].

The research presented in this paper specifically focused on landfill sites of the same type (sanitary landfills) but differing in several key aspects, such as their operational status (active or closed), waste morphology, implementation of engineered protection systems, and local environmental conditions. By comparing the results obtained from the investigated landfills with existing findings in the global literature, it was possible to gain insights into potential variations in PTEs contamination and the resulting ecological risks. Furthermore, this work represents pioneering research focused on the environmental and ecological assessment of soils affected by the operation of sanitary landfills in different regions (Poland and the Czech Republic), with a particular emphasis on the assessment of the risk of PTEs contamination.

The novelty of this study lies in the comparative analysis of different MSW landfill sites, coupled with the integration of ecological risk assessment. This approach provides valuable insights into the interplay of factors influencing PTEs contamination. In addition, the assessment of ecological risks arising from the occurrence of PTEs is necessary to protect ecosystems and human health. These assessments are essential tools for developing and implementing effective strategies to address current environmental challenges at MSW landfills.

## Material and methods

### Description of the study areas

**Fig 1:**
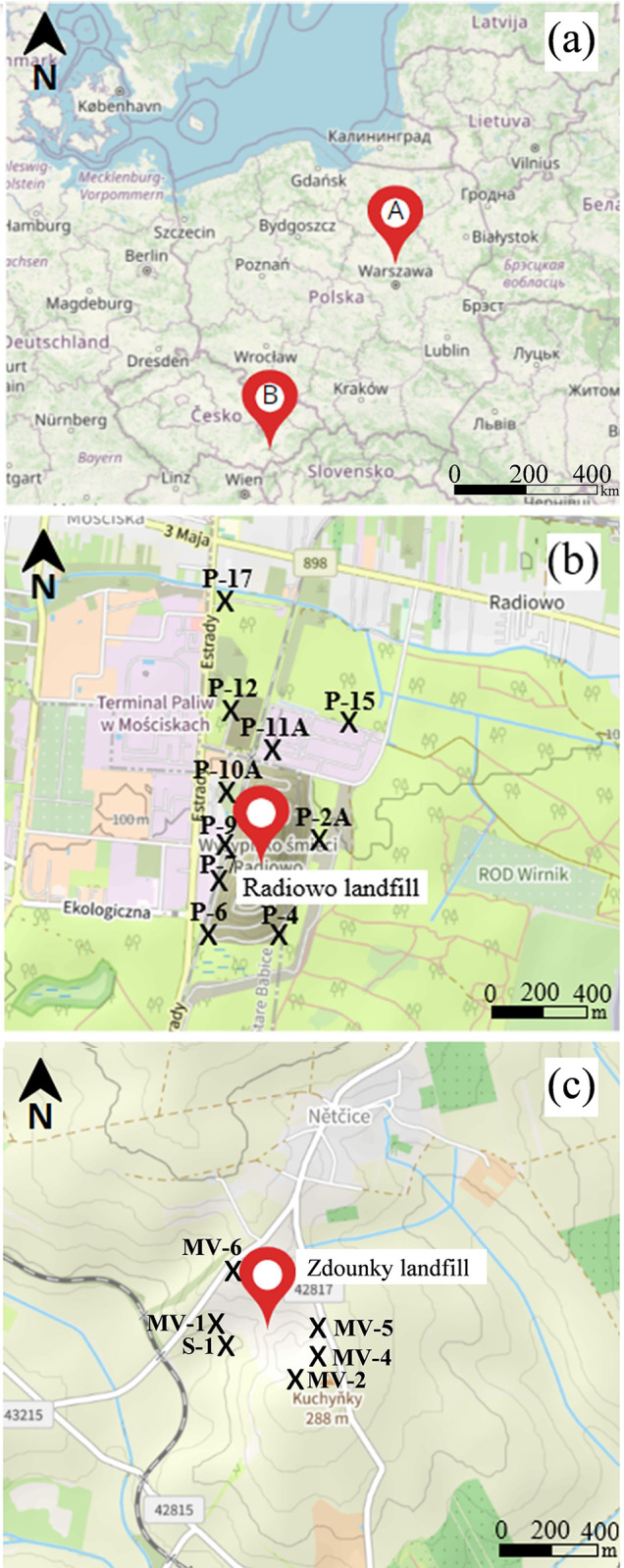
General view of the location of the Radiowo and Zdounky landfills (a); sampling points at the Radiowo (b) and Zdounky (c) sites.

The research presented focused on a detailed survey of selected MSW landfills in Poland and the Czech Republic (Fig 1A).

The base maps were obtained from OpenStreetMap and OpenStreetMap Foundation, which are available under the Open Database License [42].

### Radiowo landfill

The Radiowo landfill (52°16′37″ N, 20°52′45″ E) is located in the village of Klaudyn and part of the city of Warsaw, Poland (Fig 1B). It covers an area of about 16 ha and is 60 m height. Until 1991, unsorted MSW from Warsaw was deposited at the landfill for over 30 years. Since 1992, the landfill has been used to store screenings from the Radiowo composting plant. At that time, only waste from compost production was deposited and used to shape the landfill. The landfill is surrounded by Bemowo Forest Park, Kalinowa Łąka Flora Reserve and Łosiowe Błota Peatland Reserve. The Kampinos National Park is located about 3 km from the landfill. To the north, the landfill is bordered by a composting plant. On the western side there is a railway siding, behind which there are industrial facilities. There is a paved area about 200 m from the landfill and a fuel depot to the north-west. The Lipkowska Woda stream, a tributary of the Zaborowski Canal, flows about 400 m from the landfill. A residential area is located about 550 m from the composting plant.

Initially, the landfill was not lined at the base and no leachate collection system was designed. The first aquifer is at a depth of 0.5–2.0 m below surface level (b.s.l.) and was exposed to contamination for many years. The second aquifer is at a depth of 15–25 m b.s.l. and is isolated from the surface by layers of glacial till and locally by clay deposits [43].

Reclamation of the landfill started in 1997–1998 and the leachate collection system was installed. A vertical barrier was also constructed to prevent leachate migration [44]. In 2001, the leachate management system was extended to include pre-treated rainwater from the composting plant, which had previously been discharged into the Zaborowski Canal.

In addition, a landfill gas management system was installed, and the adjacent drainage ditches were restored. In 2016, waste disposal at the Radiowo landfill ceased and the landfill was officially closed in 2017 [45].

### Zdounky landfill

The Zdounky landfill (49°14′29.2″ N 17°18′30.3″ E) is located in the district of Kroměříž (Zlín region), Czech Republic. The landfill was constructed in 1995 as a sanitary landfill on approximately 10 ha of agricultural land and currently occupies approximately 7 ha [46]. The landfill is surrounded by agricultural land on both sides (Fig 1C). The landfill was designed to store 907,000 cubic m of waste to serve a population of 75,000. The landfill was designed to accept S-category waste, specifically sub-category S-OO3, which refers to "other waste", including biodegradable organic matter. The biogas is collected and processed in a motor-generator unit, which allows the biogas to be converted into electrical energy. In addition, part of the top of the landfill is operated as a composting plant [46].

The Zdounky landfill is an engineered facility and includes a liner system to minimize the potential for leachate migration to the surrounding environment. The liner system consists of the following layers [11]: a mineral liner of virtually impermeable soil (1 m thick); a high-density polyethylene (HDPE) geomembrane on top of the mineral liner (1.5 mm thick); a drainage layer on top of the geomembrane consisting of sand and used tires. Reclamation of the Zdounky landfill will take place between 2017 and 2019. The design life is expected to last until 2027.

### Soil sampling and analysis

Soil samples were collected, stored, transported, and prepared for analysis in accordance with regulatory requirements [47–52]. Sampling points were distributed according to their proximity to the piezometers monitoring groundwater quality at the landfill sites (Fig 1B and 1C). The geocoordinates of the sampling sites are summarized in S2 Table.

Access to the field sites for soil sampling was provided by the authorities of the Municipal Treatment Company in the capital city of Warsaw for the Radiowo landfill and by DEPOZ, spol. s r.o. for the Zdounky landfill.

For both landfills, the samples were collected from the depth 0–0.25 m b.s.l. of selected soil profiles. The granulometry of soils was analyzed following PN-EN ISO 14688–1 [53].

For the analysis of PTEs content, the soil samples were digested using a Milestone microwave oven (Start D, Italy), in accordance with Method 3051A [54]. The calibration curve method was used for the analysis of each PTE [55]. The concentrations of selected PTEs (Ni, Cd, Pb, Zn, Cu) were measured by atomic absorption spectrometry (AAS). The content of PTEs was analyzed using an iCE 3000 spectrometer (Thermo Scientific, USA). Analyses were performed in triplicate for each soil sample using analytical grade chemicals.

The pH was measured according to PN-EN ISO 10390 [56]. Soil pH was interpreted according to the classification presented by Bruce and Rayment [57].

Electrical conductivity (EC) was measured using the conductometric method. A CX-601 multimeter (Elmetron, Poland) was used for both pH and EC analysis. The interpretation of EC values was performed according to Richards [58].

### Pollution assessment and environmental standards

For the Polish landfill site, the concentrations of PTEs in soils were assessed with reference to the permissible values specified in the Regulation of the Minister of the Environment on the method of assessing the pollution of the earth’s surface [59]. For the Czech landfill, the concentrations of PTEs in soils were compared with the Decree No. 153/2016 Coll. on the establishment of details concerning the quality of agricultural land, issued by the Ministry of the Environment of the Czech Republic [60]. For comparative analysis, the standards of PTEs content in soils of selected countries were also considered.

### Determination of pollution indices

#### Geoaccumulation Index (I_geo_)

The I_geo_ was used to assess PTEs pollution in soils based on the following formula [61]:

$$I_{geo}={log}_{2}\left[\frac{C_{n}}{1.5\;B_{n}}\right]$$ (1)

where: C_n_−concentration of n-th PTE in soil referring to dry mass (DM) (mg/kg DM), B_n_−geochemical background of n-th PTE (mg/kg DM), 1.5 –background matrix correlation factor; due to possible variations in background values for a given n-th PTE in the environment and small anthropogenic influences.

#### Single Pollution Index (Pi)

The Single Pollution Index (P_i_) was applied to evaluate the degree of risk of PTEs pollution in soils, using the formula [62]:

$$P_{i}=\frac{C_{i}}{S_{i}}$$ (2)

where: C_i_−concentration of i-th PTE in soil (mg/kg DM), S_i_−background concentration of i-th PTE in soil (mg/kg DM).

Background concentrations of PTEs for I_geo_ and P_i_ calculations were assessed based on the scientific literature presenting geochemical studies from Poland and the Czech Republic [63–70].

#### Nemerow Pollution Index (PN)

The PN was applied, which considers both the maximum and average values of the single pollution index (Pi). The calculations were conducted using the formula [71]:

$$PN=\sqrt{\frac{{{{(P}_{i})}^{2}}_{max}+{{{(P}_{i})}^{2}}_{mean}}{2}}$$ (3)

where: P_i max_−maximum value of Pi for all target PTEs (mg/kg DM), Pi mean−mean value of P_i_ for all target PTEs (mg/kg DM).

#### Load Capacity of a Pollutant (PLI)

According to the formula presented by Tomlinson et al. [72], the PLI was assessed:

$$PLI={\left(\frac{C_{1}}{S_{1}}\times \frac{C_{2}}{S_{2}}\times \ldots \times \frac{C_{i}}{S_{i}}\right)}^{\frac{1}{n}}$$ (4)

### Determination of ecological risk indices

#### Risk of potentially toxic elements (ERi)

To expresses the potential ecological risk of a given PTE, the formula presented by Hakanson et al. [62] was used:

$${ER}_{i}=T_{r}^{i}\times \frac{C_{i}}{S_{i}}$$ (5)

where: *T*_*r*_^*i*^–toxic response factor of each PTE: Cd = 30, Cu = 5, Ni = 5, Pb = 5, Zn = 1.

#### Sum of individual potential risk factors (ERI)

The potential of ecological risk, regarded as a sum of individual risk factors, was calculated using the formula [62]:

$$ERI=\sum_{i=1}^{n}{ER}_{i}$$ (6)

The interpretation of calculated indices of soil pollution and ecological risk was performed based on S1 Table.

### Statistical analysis

The soil analysis results were statistically processed using Statistica 12 (StatSoft Inc., Tulsa, OK, USA). The following descriptive statistics were calculated: mean, median, minimum, maximum, standard deviation (SD), coefficient of variation (CV), skewness and kurtosis. The results were interpreted according to Rabiej [73].

Normality of the data sets was tested using the Shapiro-Wilk test. Both the Mann-Whitney U test (for non-normally distributed data) and the Student t test (for normally distributed data) were used to determine the significance of differences between the parameters analyzed.

Principal Component Analysis (PCA) was used to explain the variance in soil parameters [74, 75] and to reduce the dimensionality of the soil monitoring data for both landfills [76]. In addition, PCA was used to identify potential sources of PTEs in soils. In this analysis, variables with statistically significant relationships were identified based on r values greater than or equal to 0.5 [77].

### Analysis of PTEs spatial distribution

Spatial distribution analysis of PTEs was performed using Surfer 22 software (Golden Software Inc., Golden, USA). A variogram analysis was used to assess the spatial dependence and variability of PTE concentrations [78].

It provided valuable insights into the autocorrelation structure of the data, which aided in the selection of appropriate kriging parameters. In this study, kriging was used as a geostatistical interpolation technique to predict PTE values at locations where no samples were collected, using the spatial relationships and variability observed between the sampled data points [79].

The result was the production of surface maps visually illustrating the spatial distribution of PTEs concentrations across the study areas. These output maps provided a clear representation of the spatial patterns and variability of PTEs, facilitating the interpretation and identification of hotspots where PTEs occurred prominently [80].

## Results and discussion

### Physicochemical parameters and PTEs concentrations

No significant differences were observed between the following parameters for the two analyzed landfills: clay content (p = 0.116), gravel content (p = 0.074), pH (p = 0.745), EC (p = 0.682), Ni (p = 0.957), Zn (p = 0.175) and Cu (p = 0.478).

The soils collected from the Radiowo landfill were categorized as sandy clays, silty clays, clays, and sands. On the other hand, soils from the Zdounky landfill were classified as sandy clays, silty clays, and clayey sands. The prevalence of cohesive soils in both landfills indicates the presence of natural conditions that favor the sorption of PTEs [81] and inhibit their release to the environment [82]. This soil type may contribute to the containment of PTEs and reduce the potential for their migration and spread to the surrounding environment.

The soils at the Radiowo site had a pH range of 5.0–8.0, with an average pH of 7.3. In contrast, the soils from the Zdounky landfill had a pH range of 7.2–7.5. The neutral to alkaline pH values observed in the soils of both landfills indicate reduced mobility and reduced risk potential for the occurrence of PTEs [83]. This pH range generally indicates less favorable conditions for the leaching and mobility of PTEs in the environment, thereby contributing to a lower risk of their dispersion and potential environmental impact.

Furthermore, the soils at both the Radiowo and Zdounky landfills were found to be non-saline, with EC values lower than 2,000 μS/cm. This observation is consistent with the results of the studies by Kanmani and Gandhimathi [84] and Vijayalakshmi et al. [85], which also indicated a non-saline character for landfill soils. The non-saline nature of these soils is an important factor to consider as it may affect the movement of water and the potential for contaminants such as PTEs to leach into the surrounding environment.

**Fig 2:**
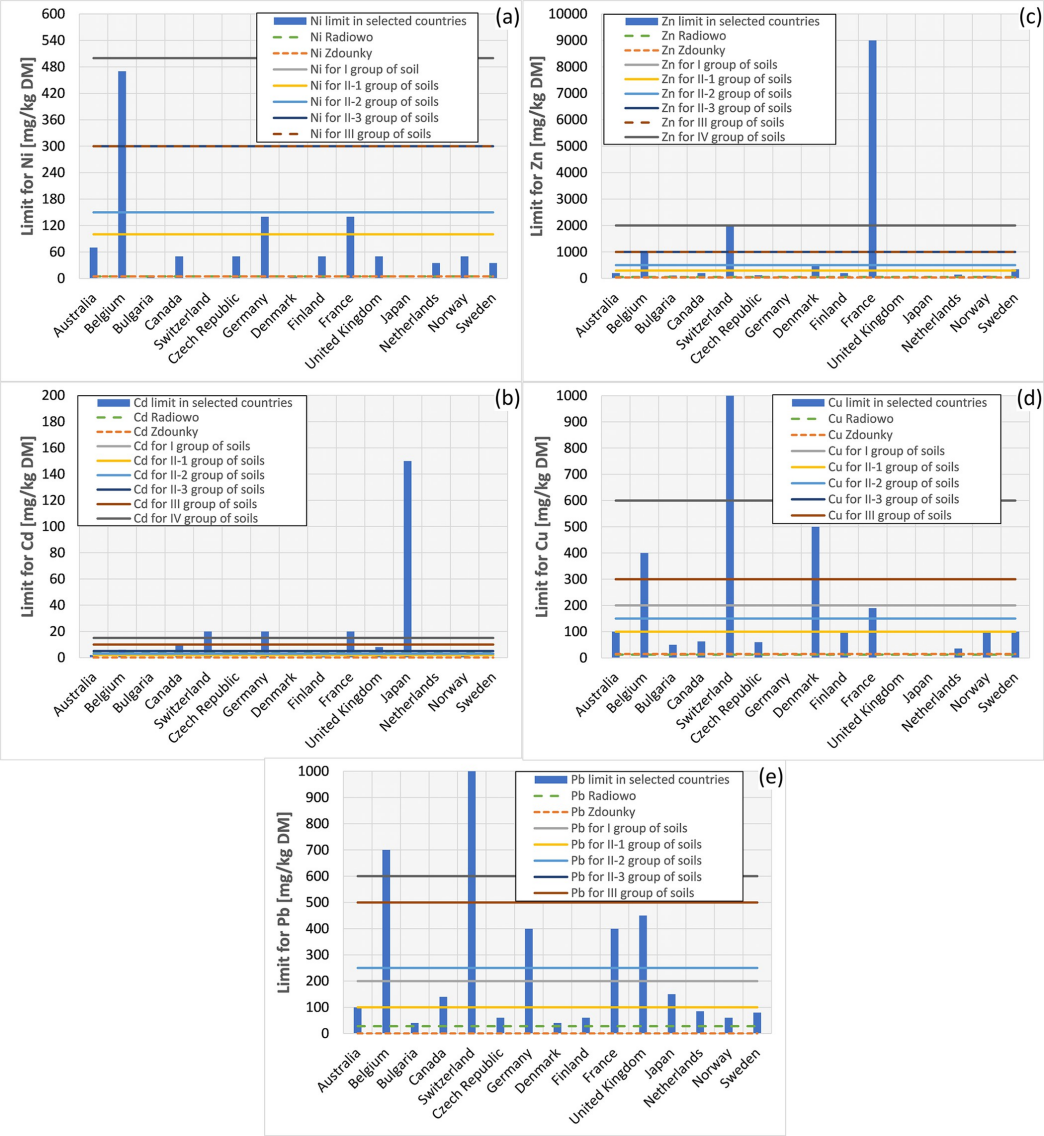
Mean concentrations of PTEs: Ni (a), Cd (b), Zn (c), Cu (d) and Pb (e) at the Radiowo and Zdounky sites in comparison with the limit values assigned to different soil groups and selected countries.

Analysis of Ni concentrations in the soils of both landfill sites indicates that the average levels are within the permissible limits set by the environmental legislation of Poland and the Czech Republic. Furthermore, these concentrations meet the criteria set by other countries (Fig 2A).

The mean concentrations (3.49 mg/kg DM) of Cd at the Radiowo site exceeded the established limits for Group I (2 mg/kg DM), as well as for Groups II-1 (2 mg/kg DM) and II-2 (3 mg/kg DM) (Fig 2B). The higher Cd concentrations near the landfill could be due to its water solubility, which allows it to enter the soil via water pathways [86].

In addition, the increased mobility of Cd compared to other PTEs in soil may be due to factors such as competition and ligand-induced desorption [87]. However, previous research suggests that even with this increased potential for mobility, Cd does not pose a threat to groundwater. This is supported by the favorable groundwater chemical status observed in the vicinity of the Radiowo site [45]. These results strongly suggest that site conditions are favorable for effective sorption of Cd within the subsoil of the Radiowo site.

In the case of Cd in the soil of the Zdounky area, it was observed that the measured concentrations were well below the threshold values (Fig 2B). Moreover, these measured concentrations remained below the threshold values established for different soil use categories, including residential areas (group I); arable land, orchards, meadows, and pastures, depending on soil characteristics (groups II-1, II-2, II-3); and forests, wooded and vegetated areas, wasteland, historical sites, and ecological areas (group III). This pattern also extended to industrial, mining and transport zones (Group IV). A similar scenario unfolded for Zn concentrations (Fig 2C), where the levels detected at the investigated landfills were within the permissible limits. Furthermore, the concentrations of Cu (Fig 2D) and Pb (Fig 2E) measured at the Radiowo and Zdounky sites were well below the thresholds set by the environmental legislation of various countries.

The results presented in this study differ from some of the results reported in the literature, such as those presented by Odom et al. (2021) [88]. According to them, the concentrations of PTEs (Cd, Cu, Fe and Zn) in the soil studied at the Dompoase landfill site exceeded the Environmental Protection Agency and World Health Organization guidelines (except for Ni, which was detected below the limit of detection).

The reduced PTE concentrations may be attributed to the successful application of advanced landfill design and sustainable WM practices [46, 89]. Engineered barrier systems, consisting of impermeable liners and leachate collection systems, effectively limit the movement of PTEs beyond the boundaries of the landfill [11]. In addition, the composition of the waste materials deposited in these landfills can play a critical role in reducing PTE levels [90]. A waste stream consisting primarily of materials with inherently low concentrations of PTEs would result in reduced concentrations within the landfill environment. In conjunction with this, WM practices, including sorting and recycling, could effectively remove waste items rich in PTEs from the disposal process [91]. This approach in turn reduces the overall PTE content entering the landfill.

In addition, the inherent dynamics of PTEs within the landfill environment should also be considered. Over time, PTEs can undergo various chemical transformations, including weathering, precipitation and complexation with other substances present in the soil [92]. These processes can lead to the immobilization of PTEs, thereby reducing their mobility and bioavailability within the soil matrix [93].

Natural attenuation processes, such as adsorption to soil particles [94, 95] and interactions with microbial communities, may also contribute to the observed low concentrations [96]. In addition, the collection and treatment of leachate prevents the release of PTEs into the surrounding soil and water, further contributing to the overall reduction in concentrations [97].

A combination of engineering solutions, WM strategies, environmental regulations and natural processes are likely to be responsible for the low concentrations of PTEs at the Radiowo and Zdounky landfills. This phenomenon highlights the potential effectiveness of sanitary landfill practices in mitigating the environmental impact of PTE contamination [98]. Further investigation, including detailed geochemical and microbial analyses [99], would shed light on the complex interplay of these factors and provide a full understanding of the observed results.

### Pollution indices

**Fig 3:**
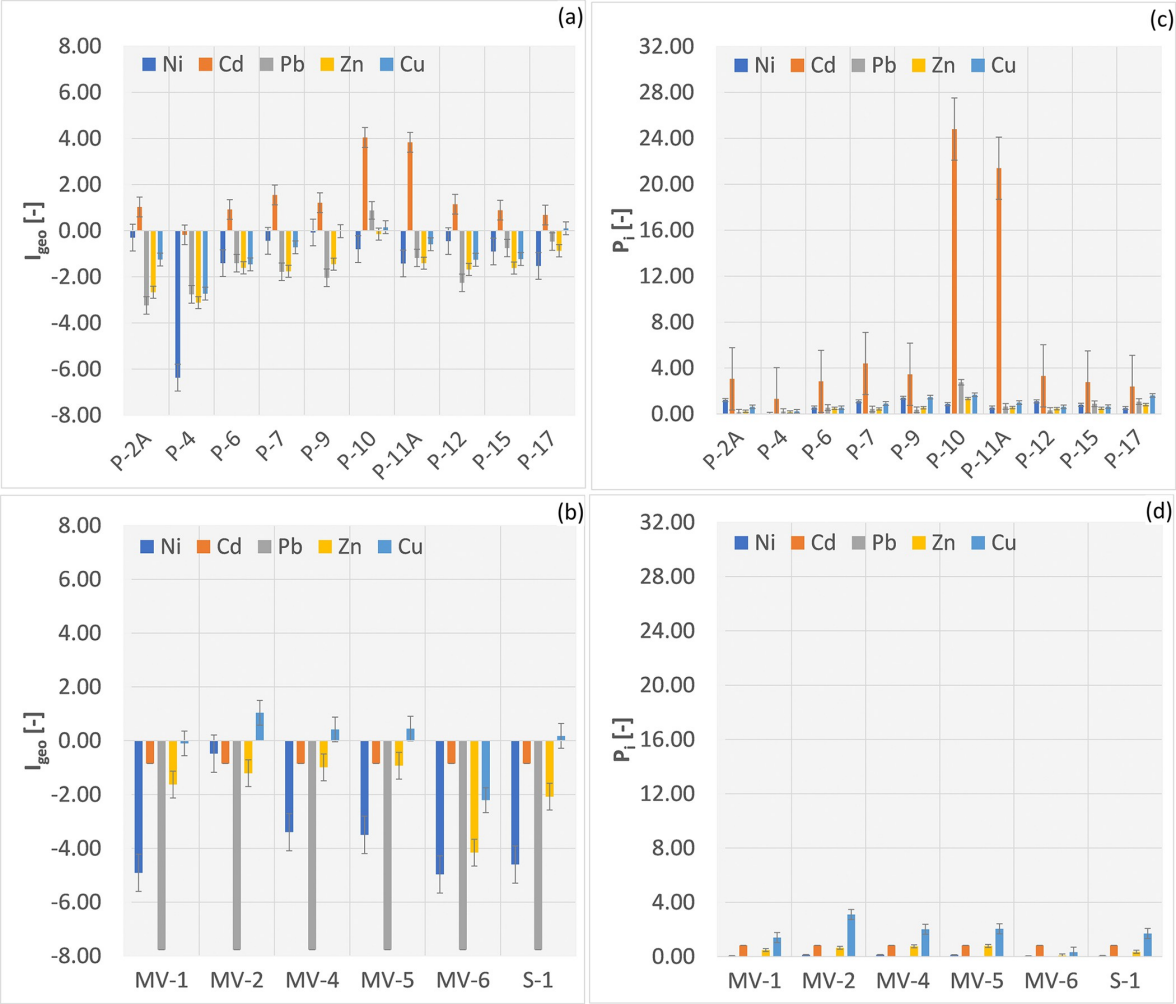
I_geo_ and P_i_ of PTEs in soils of the Radiowo (a, c) and Zdounky (b, d) landfills.

When assessing the contamination of the Radiowo landfill using I_geo_, it was found that points P-10 and P-11 indicate heavy contamination, especially for Cd (Fig 3A). On the other hand, for the remaining points, the Cd concentrations in the soils indicate an unpolluted to moderately polluted status.

Regarding I_geo_ for Ni, Pb, Zn and Cu, it has been indicated that the soils are classified as unpolluted. For the Zdounky area, the majority of the soils are classified as unpolluted (Fig 3B), except for points MV-2, MV-4, MV-5 and S-1, which indicate unpolluted to moderately polluted status of the soils in terms of Cu. For comparison, Ahmad et al. [97] found that the I_geo_ in industrial areas indicated significant soil contamination for all PTEs except Cu (≈ 3.0), Cd (≈ 3.0) and Pb (2.97), classifying the contamination level as moderate. Their study showed a significant ecological risk specifically related to Cd and Hg in the soil, while the contributions of Cr, Cu, As and Pb were associated with a low ecological risk. Low I_geo_ values calculated for PTEs in soils are typical for sanitary landfills [47].

Single pollution indices calculated for the Radiowo landfill indicate that high contamination of Cd exists in points P-10 and P-11 (P_i_ > 6). Significant Cd contamination is observed in points P-2A, P-7, P-9 and P-12, while moderate contamination occurs in points P-4, P-4, P-15 and P-17 (Fig 3C). This has also been demonstrated by Singh and Chandel [100] who found that Cd is the most polluting and mobile PTE in the Mumbai landfill site in India. In addition, studies by Sabet Aghlidi [39] have shown that industrial activities and agronomic practices, including the use of livestock manure and especially phosphorus fertilizers, contribute to elevated Cd concentrations in soils.

For the Zdounky landfill, only point MV-2 was found to be significantly contaminated in terms of Cu occurrence. It was observed that points MV-1, MV-4, MV-5 and S-1 show a moderate level of contamination. For the remaining PTEs, the Pi < 1 indicates the absence of contamination (Fig 3D).

**Fig 4:**
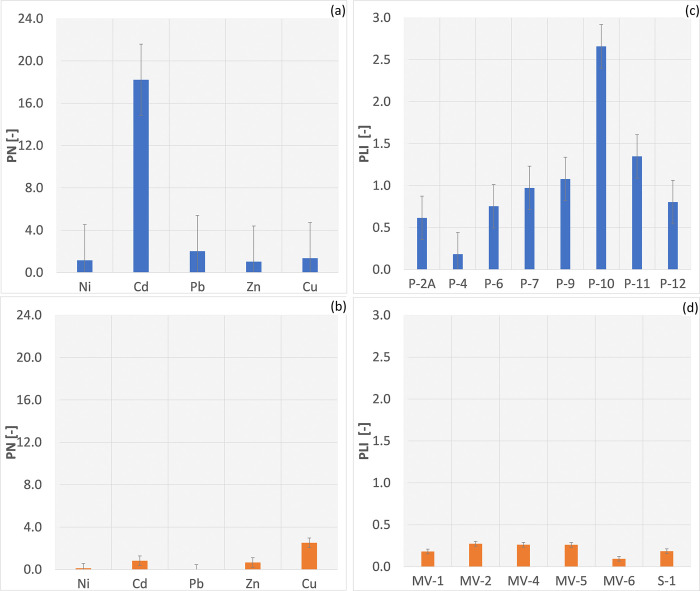
PN and PLI in the soils of the Radiowo (a, c) and Zdounky (b, d) landfills.

According to the PN, an assessment of PTEs pollution in the Radiowo area showed a significant level of pollution for Cd with a PN value of 18.22. Moderate pollution was observed for lead (Pb), while slight pollution was identified for Ni, Zn, and Cu (Fig 4A). In the study by Wu et al. [101], the risk assessment analysis at construction landfills heighted that Cd and Mn pose the most significant environmental risk due to their elevated effective content, bioavailability, and mobility. Similar findings were presented by Zhou et al. [102], who identified Cd, As, and Hg as the major contaminants in the ecological risk at the landfill site in Lhasa, Tibet.

For Cu in the Zdounky area, a warning level of contamination was assigned, but on the other hand, the levels of Ni, Pb and Zn in the Zdounky area suggest that the soils can be classified as safe (Fig 4B).

The PTEs measured for the Radiowo area indicates a high level of contamination at point P-10 (Fig 4C), which, as previously shown, can be explained by the high Cd concentrations. Moderate levels of pollution were observed at points P-9 and P-11. The remaining points of the Radiowo landfill, as well as the Zdounky site (Fig 4D), show low levels of contamination.

### Ecological risk

**Fig 5:**
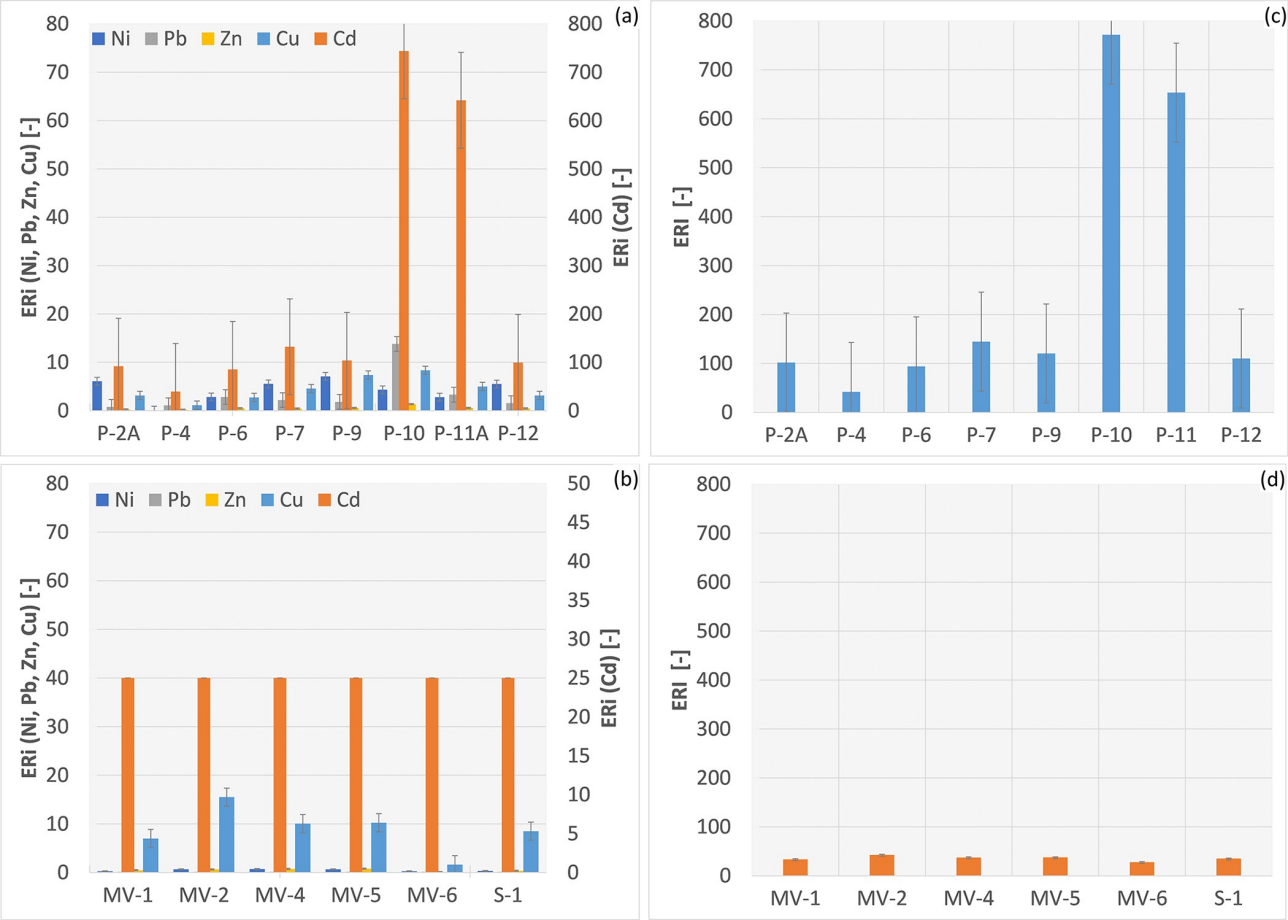
ERi and ERI in the Radiowo (a, c) and Zdounky (b, d) landfills.

In the Radiowo area, the ecological risk assessment based on high Cd concentrations showed an extremely high ecological risk at points P-10 and P-11 (Fig 5A).

These sites have high Cd concentrations. In addition, points P-2A, P-6, P-7, P-9 and P-12 were classified as moderate ecological risk. In contrast, point P-4 was classified as low ecological risk. For the Zdounky area, all ERi values were less than 40, indicating a low ecological risk from PTEs at this site (Fig 5B).

Based on the analysis of the ERI, a high ecological risk was identified for points P-10 and P-11 at the Radiowo site (Fig 5C). In contrast, low ecological risk was assigned to the remaining points tested at the Radiowo site. Similarly, at the Zdounky site, ERI values indicated a low ecological risk (Fig 5D). Similarly, in the study presented by Karimian et al. [103], the ER_i_ values were lower than 40, indicating a low level of contamination in the landfill areas, while a moderate ecological risk was observed for Cd (ER_i_ = 75.4).

### Spatial distribution of PTEs

**Fig 6:**
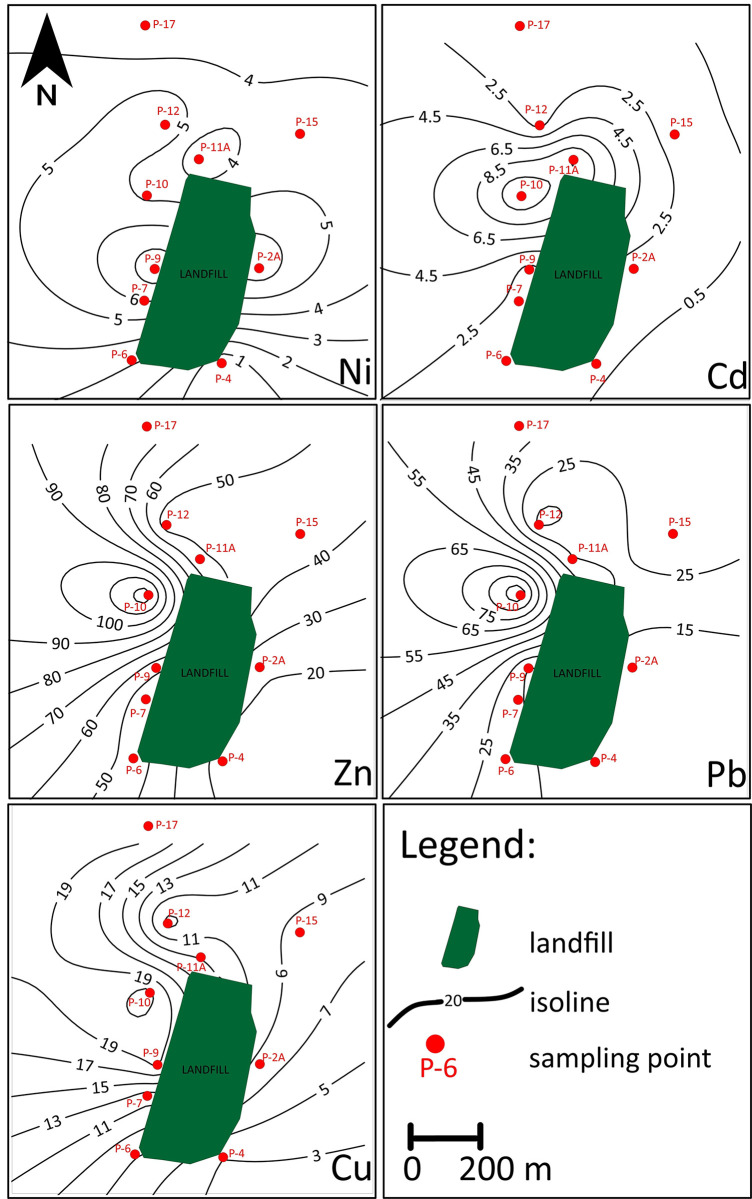
Spatial distribution of PTEs (mg/kg DM) in soils at the Radiowo site.

**Fig 7:**
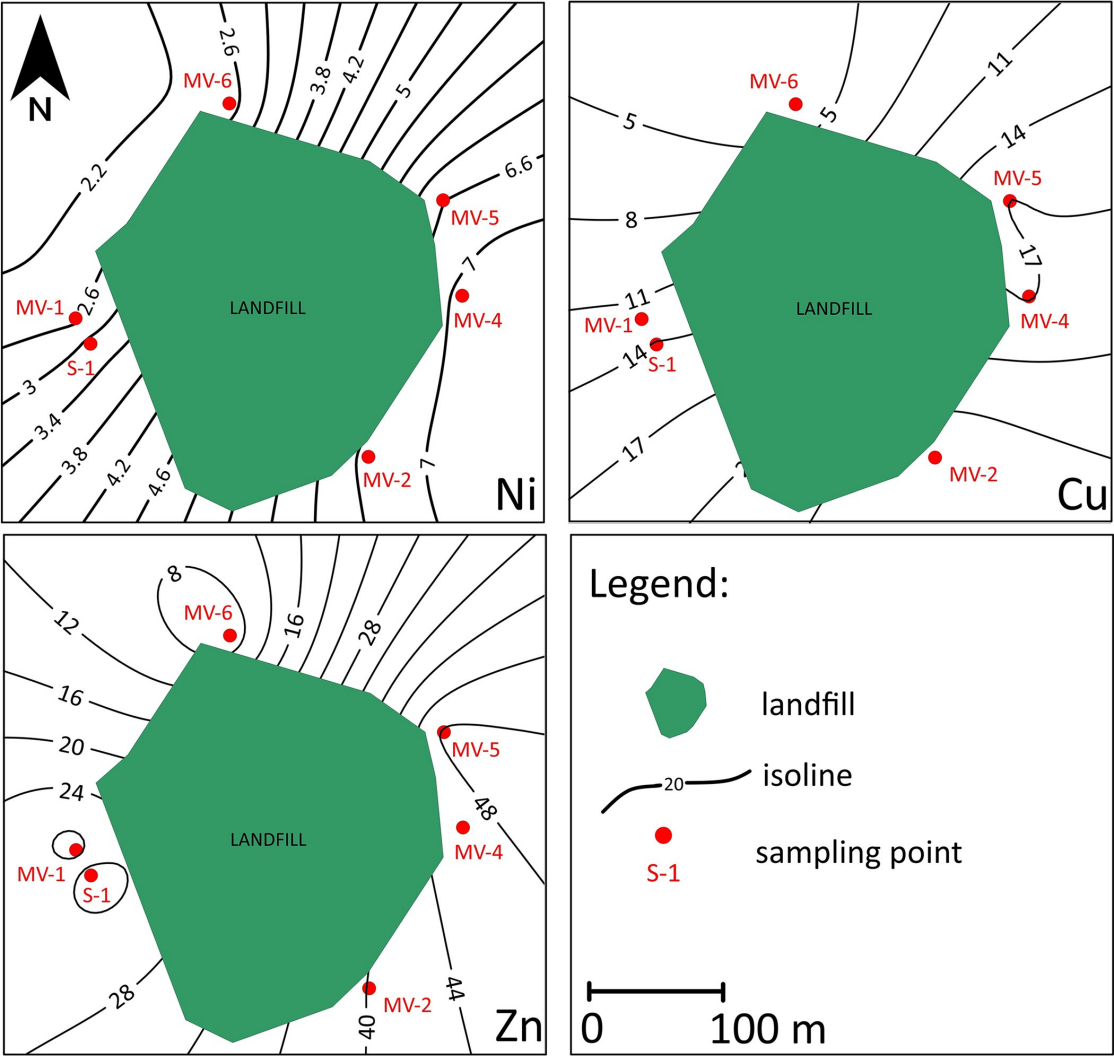
Spatial distribution of PTEs (mg/kg DM) in soils at the Zdounky site.

Observation of the spatial variability for each of the PTEs indicated that the concentrations of these elements are not uniformly distributed across the Radiowo (Fig 6) and Zdounky (Fig 7).

The excessive concentrations of Cd, Zn, Pb and Cu in the vicinity of point P-10 near the Radiowo landfill (Fig 6) indicate the presence of a potential source of PTEs contamination in this area. The elevated levels of these PTEs could be attributed to the nearby metallurgical production activities, which may contaminate the surrounding environment, including the soil. The study by Wieczorek et al. [104] supports the notion that the excessive concentration of Cd and Pb in soils may be due to the influence of mining and metallurgical activities involving the extraction and processing of metals. However, further research is needed to establish a direct cause-effect relationship between metallurgical production and the observed PTEs contamination. The presence of a high level of Cd measured at point P-11, located in the vicinity of a waste treatment plant, suggests a possible source of pollution related to the operation of this plant.

The analysis of the spatial distribution of PTEs in the Zdounky area (Fig 6) showed that the highest concentrations were observed on the eastern side, especially in the vicinity of agricultural areas. This finding suggests a possible link between agricultural activities (application of fertilizers and pesticides containing PTEs) and the elevated levels of PTEs in the soil [105, 106].

Spatial variability in the distribution of PTEs can result from several factors, including natural processes, anthropogenic activities, and local soil characteristics. According to Obiri-Nyarko et al. [107], variations in the spatial distribution and heterogeneity in the type and amount of PTEs present at a given site may result from different sources of these PTEs. Understanding the spatial variability of PTEs in soils is of paramount importance for effective environmental management and the formulation of remediation strategies. This understanding facilitates targeted sampling and monitoring to identify areas of potential contamination. In addition, understanding the patterns of spatial distribution can contribute to the development of predictive models aimed at estimating PTE concentrations in areas where sampling has not been carried out [108].

### Interrelationships between PTEs in soils

**Fig 8:**
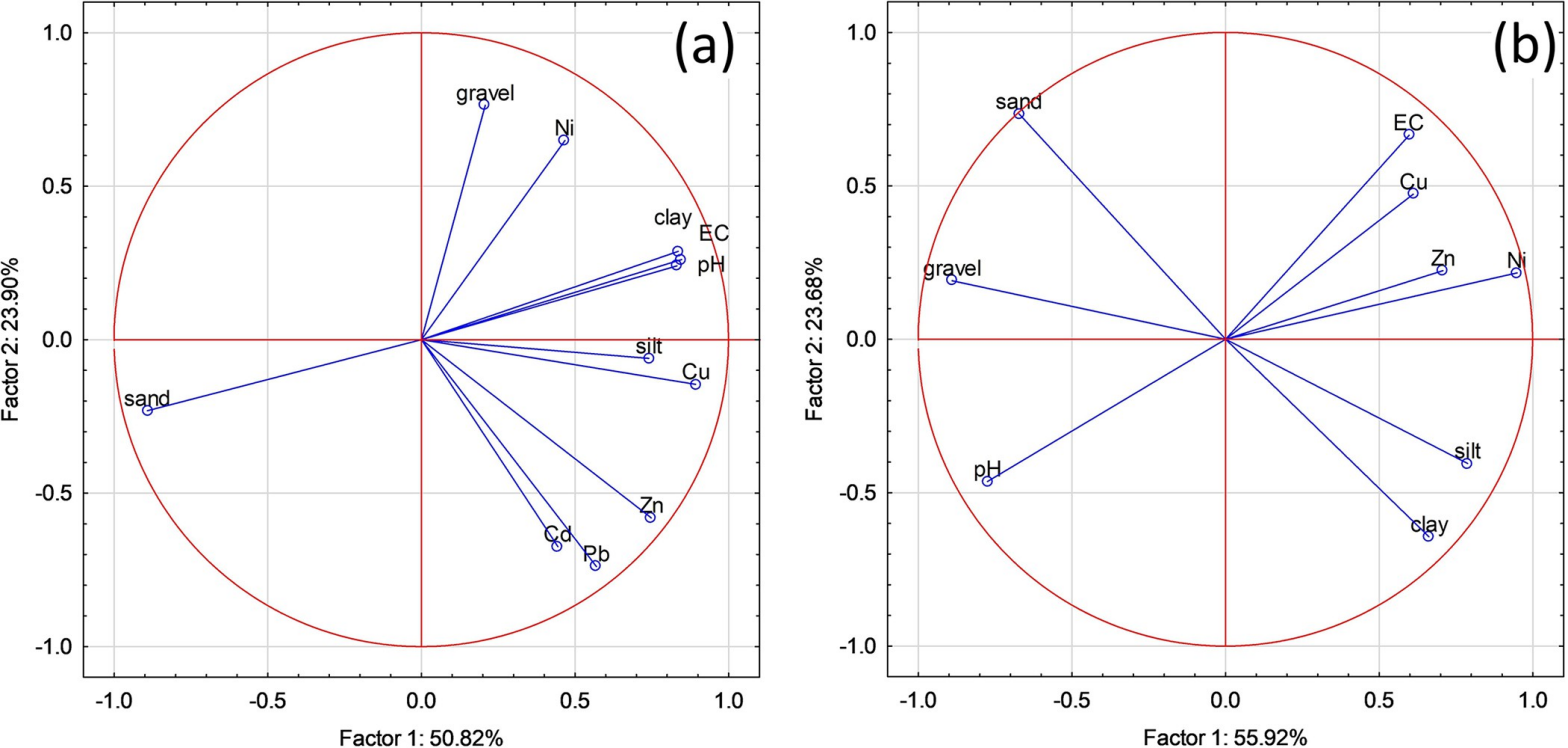
The results of PCA performed for the Radiowo (a) and Zdounky (b) landfills.

PCA was conducted for the Radiowo landfill site, resulting in the transformation of soil parameters into three principal components (PCs) that collectively account for 87.12% of the total variance. The PC1, representing 50.81% (Fig 8A) of the variance, shows notable positive loadings for pH, EC, Cu, and the clay fraction.

Conversely, the sand fraction has a strong negative loading in PC1. This suggests that these variables are closely related and probably have a common underlying influence. PC2, which explains 23.90% of the variance, is significantly related to the gravel fraction. PC3, which explains 12.41% of the variance, shows a moderate positive loading for Ni and a moderate negative loading for the silt fraction.

Moderate loadings of Pb, Zn and Cu within PC1 suggest a possible common origin for these PTEs. This finding is consistent with previous research by Obiri-Nyarko et al. [107] who highlighted strong positive correlations between Cu, Pb and Zn, indicating their common association and likely common source at the landfill. In addition, Wieczorek et al. [104] confirmed that sand, silt, and clay fractions together with Pb and Cd concentrations often formed PC1. This agreement with the results of the present study confirms the consistency of this pattern, except for the significant contribution of Cd to PC2 in the present study. The grouping of certain PTEs within the same component suggests possible common origins, echoing similar observations in the literature.

The PCA analysis performed for the Zdounky landfill revealed three PCs that together account for a substantial 95.16% of the total variance. PC1, which captures 55.92% of the total variance, shows pronounced negative loadings for pH, sand, and gravel fraction (Fig 8B). In contrast, positive loadings in PC1 are assigned to EC, Ni, Zn, Cu, and the silt fractions. PC2 accounts for 23.68% of the total variance and shows a moderate positive loading related to EC and the sand fraction. A moderate negative loading is observed for the clay fraction within PC2. PC3 contributes 15.57% of the total variance and shows moderate negative loadings for Zn and Cu. The moderate influence of PTEs on PC1 suggests a common source and similar properties among them. Furthermore, the parallel pattern of PTEs within the analyzed soils strongly suggests a common source influencing their presence [109].

### Implications for addressing environmental risk and remediation strategies

PTEs pose significant risks to human health and the environment, particularly in landfills where their presence can lead to contamination of soil and groundwater. It was also evidenced by Islamd et al. [110] who indicated that the disposal of waste associated with urban activities is the main contributor to the presence of PTEs in soils across various types of land use.

This study presents a comprehensive approach to identify and assess the occurrence of PTEs in landfills, highlighting their potential hazards and impacts on soil quality. By analyzing PTE concentrations and comparing them with environmental limit values, this research provides valuable insights into the effectiveness of WM practices and the need for sustainable waste treatment processes.

The transport and disposal of waste, including MSW, has raised concerns about the release of PTEs into the environment. Studies have demonstrated the negative impact of landfills on soil quality and groundwater due to leaching of PTEs. This paper addresses the urgent need to understand and manage PTE contamination in landfills to protect public health and environmental integrity [90]. The release of PTEs from waste materials in landfills can also lead to ecotoxicity [111]. The ecotoxicological risk of PTE contamination from MSW landfills was also highlighted by Alghamdi et al. [112] who demonstrated the negative impact of landfills on the quality of nearby soils and groundwater.

In relation to the study presented, it was found that cohesive soils can be a dominant factor in retaining PTEs and mitigating their potential migration. At the same time, unfavorable pH conditions were identified as a significant deterrent to leaching, thereby reducing the environmental impact. The relationships between fractional content and PTE distribution were also described by Caporale et al. [113] who found that PTEs tended to accumulate in the finer particle size fractions. A contrasting assessment was provided by Minkina et al. [114] who indicated that the patterns of soil contamination are strongly influenced by the local atmospheric circulation, while the characteristics of the soils within the study area play a secondary role.

The concentrations of PTEs were generally within acceptable limits due to well-designed landfills, effective WM practices and natural attenuation processes. However, non-uniform concentrations of PTEs were observed, possibly influenced by nearby industrial activities and agricultural practices. Despite generally acceptable PTE concentrations, localized hotspots were identified that require continuous monitoring and intervention. In addition, in the study areas with elevated PTEs levels, restrictions may be placed on certain types of development or specific precautions may be enforced to ensure the safety of residents and workers.

The study by Agyeman et al. [115] also demonstrated that the mapping of PTE occurrence hotspots helps to identify areas requiring immediate remediation. Furthermore, low-density mapping of contaminant occurrence, supported by detailed statistical analysis in an area of multiple contamination sources, is able to capture the major contamination sources, trends and geochemical processes in relation to sensitive receptors [116].

PTEs in soil do not only originate from landfilling. Concentrations of PTEs are elevated in areas with industries such as chemicals, metals/electronics, manufacturing, and other industrial sectors [117]. The continuous application of phosphorus fertilizers can also increase PTE levels in soils [115]. Soils can be furthermore affected by PTEs from power plants [114] and coal mining [116], increasing the risk to public health in the vicinity of these facilities.

Regular assessments of PTE levels and comparisons with environmental thresholds provide a solid basis for ongoing, long-term environmental monitoring. This continuous monitoring is essential to track changes over time, to evaluate the effectiveness of mitigation measures implemented, and to provide early warning in case of resurgence of PTEs concentrations. In particular, continuous monitoring of PTEs should be considered to reduce exposure to PTEs [118].

Understanding and mitigating risks in identified hotspots helps to maintain ecological functions, including water purification, soil fertility and biodiversity support. By identifying areas of elevated ecological risk, action can be taken to minimize potential health risks, particularly where hotspots are close to residential areas. In response to the growing need to address PTE contamination, remediation methods should be developed using mechanical, physicochemical, or biological technologies [96, 119].

## Conclusions

Compared to the current literature, this study advances the knowledge on the likely contamination of waste disposal areas (landfills) through a detailed spatial analysis of PTEs distribution and ecological risk. The identification of specific risk points at the Radiowo and Zdounky sites contributed to the understanding of potential hazards in landfill environments. However, it should be noted that the spatial analysis may not cover all potential sources of PTEs and that the scope of the study is limited to specific landfill sites.

Future research should consider expanding the geographic scope to include a wider range of soil types and land uses. Continuous monitoring of metallurgical activities and agricultural practices, coupled with targeted interventions, should be proposed. In addition, the establishment of buffer zones and regular maintenance programs can address emerging environmental problems in a timely manner. Future studies should also address the long-term effects of PTEs on soil toxicity and biological productivity as well as their interrelationships with contaminants of emerging concern (CECs), and assess the effectiveness of different mitigation strategies in different environmental contexts.

## Supporting information

S1 Table Classification of soil pollution and ecological risk according to various indices. (DOCX)

S2 Table Geo-coordinates of sampling locations. (DOCX)
