# Supplementary material for: Potentially toxic elements (PTEs) and ecological risk at waste disposal sites: An analysis of sanitary landfills
Source: PLoS One. 2024 May 17;19(5):e0303272. doi: 10.1371/journal.pone.0303272 (PMC11101111; doi:10.1371/journal.pone.0303272)
Supplement: S1 Table — (DOCX) [file pone.0303272.s001.docx]

**S1 Table. Classification of soil pollution and ecological risk according to various indices.**

| **Classification** | **Interpretation** |
| --- | --- |
| *I_geo_* | |
| I_geo_ ≤ 0; class 0 | Unpolluted |
| 0 < I_geo_ ≤ 1; class 1 | From unpolluted to moderately polluted |
| 1 < I_geo_ ≤ 2; class 2 | Moderately polluted |
| 2 < I_geo_ ≤ 3; class 3 | From moderately to strongly polluted |
| 3 < I_geo_ ≤ 4, class 4 | Strongly polluted |
| 4 < I_geo_ ≤ 5; class 5 | From strongly to extremely polluted |
| I_geo_ > 5; class 6 | Extremely polluted |
| *P_i_* | |
| Pi < 1 | Low contamination |
| Pi > 1 | Pollution exists |
| 1 ≤ Pi < 3 | Moderate contamination |
| 3 ≤ Pi < 6 | Considerable contamination |
| Pi ≥ 6 | High contamination |
| *PN* | |
| PN ≤ 0.7 | Safe |
| 0.7 < PN ≤ 1 | Warning |
| 1 < PN ≤ 2 | Light pollution |
| 2 < PN ≤ 3 | Moderate pollution |
| PN > 3 | Heavy pollution |
| *PLI* | |
| PLI ≤ 1 | Low level of pollution |
| 1 < PLI ≤ 2 | Moderate level of pollution |
| 2 < PLI ≤ 5 | High level of pollution |
| PLI > 5 | Extremely high level of pollution |
| ER_i_ | |
| ER_i_ < 40 | Low ecological risk |
| 40 ≤ ER_i_ < 80 | Moderate ecological risk |
| 80 ≤ ER_i_ < 160 | Strong ecological risk |
| 160 ≤ ER_i_ < 320 | Quite strong ecological risk |
| ER_i_ ≥ 320 | Extremely strong ecological risk |
| *ERI* | |
| ERI <150 | Low ecological risk |
| 150 ≤ ERI < 300 | Moderate ecological risk |
| 300 ≤ ERI < 600 | Strong ecological risk |
| ERI > 600 | Quite strong ecological risk |
