# Supplementary material for: Potentially toxic elements (PTEs) and ecological risk at waste disposal sites: An analysis of sanitary landfills
Source: PLoS One. 2024 May 17;19(5):e0303272. doi: 10.1371/journal.pone.0303272 (PMC11101111; doi:10.1371/journal.pone.0303272)
Supplement: S2 Table — (DOCX) [file pone.0303272.s002.docx]

**S2 Table. Geo-coordinates of sampling locations.**

| **Point** | **Landfill** | **Geographic coordinates** | |
| --- | --- | --- | --- |
| P-2A | Radiowo | 20°52’54.8’’ E | 52°16’38.1″ N |
| P-4 |  | 20°52’48.2’’ E | 52°16’27.8″ N |
| P-6 |  | 20°52’34.4’’ E | 52°16’27.9″ N |
| P-7 |  | 20°52’35.9’’ E | 52°16’34.7″ N |
| P-9 |  | 20°52’37.4’’ E | 52°16’37.7″ N |
| P-10A |  | 20°52’36.6’’ E | 52°16’45.6″ N |
| P-11A |  | 20°52’45.0’’ E | 52°16’49.6″ N |
| P-12 |  | 20°52’39.0’’ E | 52°16’53.7″ N |
| P-15 |  | 20°53’03.8’’ E | 52°16’51.9″ N |
| P-17 |  | 20°52’35.0’’ E | 52°17’04.2″ N |
| MV-1 | Zdounky | 17°18’21.3’’ E | 49°14’28.0″ N |
| MV-2 |  | 17°18’32.2’’ E | 49°14’24.2″ N |
| MV-4 |  | 17°18’36.4’’ E | 49°14’27.8″ N |
| MV-5 |  | 17°18’35.4’’ E | 49°14’30.7″ N |
| MV-6 |  | 17°18’27.1’’ E | 49°14’33.3″ N |
| S-1 |  | 17°18’21.6’’ E | 49°14’27.1″ N |
